# Adsorption of Pb^2+^ and Cd^2+^ from Aqueous Solutions by Porous Carbon Foam Derived from Biomass Phenolic Resin

**DOI:** 10.3390/ijms26157302

**Published:** 2025-07-28

**Authors:** Jianwei Ling, Yu Gao, Ruiling Wang, Shiyu Lu, Xuemei Li, Shouqing Liu, Jianxiang Liu

**Affiliations:** 1 College of Ecology and Environment (College of Wetlands), Southwest Forestry University, Kunming 650224, China; 2 College of Soil and Water Conservation, Southwest Forestry University, Kunming 650224, China; 3 College of Biological and Food Engineering, Southwest Forestry University, Kunming 650224, China; 4 College of Materials and Chemical Engineering, Southwest Forestry University, Kunming 650224, China

**Keywords:** modified carbon foam, silane coupling agent, adsorption, lead, cadmium, removal mechanism

## Abstract

Due to its lightweight and superior adsorption properties, carbon foam is frequently employed for the removal of heavy metal pollutants from aqueous solutions. In this study, a novel modified carbon foam (M-CF) was successfully synthesized for the effective removal of Pb^2+^ and Cd^2+^ from water. The synthesis involved partially substituting phenol with the liquefaction product of bamboo powder, followed by modification with a silane coupling agent (KH560) and foaming with n-hexane-loaded activated carbon (H/AC). The prepared carbon foam was comprehensively characterized, and its adsorption performance and mechanism for Pb^2+^ and Cd^2+^ in aqueous solution were investigated. The results showed that M-CF possessed a uniform and well-developed spherical pore structure and demonstrated excellent removal capacity for Cd^2+^ and Pb^2+^. The adsorption process conformed to the Sips isotherm model and the pseudo-second-order kinetic equation, with maximum adsorption capacities of 22.15 mg·g^−1^ and 61.59 mg·g^−1^ for Cd^2+^ and Pb^2+^, respectively. Mechanistic analysis revealed that the removal of Cd^2+^ and Pb^2+^ was a result of the synergistic effect of physisorption and chemisorption, accompanied by complexation. Furthermore, precipitates formed during the adsorption process were found to be mainly composed of hydroxides, carbonates, and PbS. This research demonstrates the efficacy of carbon foam prepared from bamboo powder waste as a partial phenol substitute for the efficient removal of Pb^2+^ and Cd^2+^ from water, thus expanding the preparation pathways for novel heavy metal adsorption materials.

## 1. Introduction

Over the past few decades, heavy metal pollution has emerged as a major environmental concern. These pollutants are ubiquitous in soil, water, and air, posing a significant threat to human health [1]. During industrial production, highly soluble heavy metals are released into the environment, potentially contaminating surface water through river runoff or groundwater infiltration [2]. Common heavy metal pollutants include Cd, Zn, Cu, and Pb, among which Cd and Pb are among the most severe global pollutants. Long-term excessive lead intake induces multisystem toxicity in humans, with the kidneys being one of the primary target organs [3]. Cd, a persistent and difficult-to-degrade heavy metal, primarily damages the kidneys, lungs, and nervous system [4]. Despite extensive research on remediation of Cd and Pb pollution in water, such as phytoremediation [5] and chemical remediation methods [6], these approaches are often hindered by limitations including poor remediation efficiency, prolonged treatment duration, and high costs. Therefore, the efficient and economical removal of Cd and Pb from water remains a significant challenge.

In recent years, the utilization of agricultural and forestry waste to produce various derived materials [7,8,9,10] has garnered significant attention. This attention stems partly from the recognition of biomass as the only viable renewable alternative to fossil fuels [11], as well as recognition of its low cost and abundant availability. According to estimates, approximately 20.3 billion tons of agricultural waste were generated globally in 2019 alone, including 4.3 billion tons of crop straw and 16 billion tons of animal manure [12]. The vast reserves of these waste materials, combined with the gradual depletion of fossil fuels, poses two interconnected challenges for the international community [13]. Consequently, efforts have intensified to convert such waste into value-added products, including biofuels [14], particleboard [15], water purification materials [16], electrode materials, catalyst supports, biosorbents [17], and polyurethane foams [18], to meet escalating global demand.

As a common type of biomass waste, bamboo waste has also been successfully recycled and reused through thermo-chemical and biotechnological approaches [19]. Among these, combustion, pyrolysis, and gasification are key thermo-chemical routes for converting bamboo waste into value-added products such as biofuels and energy. Adeniyi et al. [20] investigated the thermochemical conversion of bamboo culms into biochar, achieving energy and material recovery from waste biomass. Esfahani et al. [21] utilized waste fibers from the bamboo construction industry to prepare membrane materials via phase inversion using extracted cellulose. Furthermore, bamboo waste can be processed into biomass-derived hard carbon anodes through a secondary sintering method, facilitating resource recycling [22]. In addition to these materials, bamboo waste can be converted into biofuels using biocatalysts and microorganisms with biocatalytic activity, thereby achieving value-added recycling of bamboo waste [23].

Carbon foam is a novel carbon material characterized by a three-dimensional honeycomb-like structure, synthesized via foaming and carbonization techniques using high-carbon organic compounds (e.g., coal, pitch, resins, or biomass) as carbon sources [24]. Its internal porous structure is typically categorized as either reticulated or cellular, with cellular carbon foam exhibiting continuous pore wall structure. This unique architecture imparts a large surface area while retaining inherent carbon material properties, such as a low density, high thermal stability, and hydrophobicity [25]. Due to these advantageous characteristics, carbon foam has been widely applied as a versatile material in lithium-ion battery electrodes [26], CO_2_ capture [27], photocatalysis [28], electromagnetic interference shielding [29], and heavy metal adsorption from wastewater [30]. In addition to its unique structure, the surface of porous carbon foam’s pore walls features a large number of nanoscale micropores, resulting in a relatively high specific surface area. Compared to activated carbon, its lower cost makes it a promising candidate for development as a commercial adsorbent material [31]. However, conventional carbon foam often fails to meet the stringent requirements of specialized industrial applications, particularly due to its limited adsorption capacity. Consequently, researchers have increasingly focused on enhancing its performance through modification strategies, with modified carbon foams demonstrating superior heavy metal adsorption capabilities. For instance, Zhang et al. [32] incorporated ZnO particles into carbon foam, achieving substantial adsorption capacities for Pb^2+^, Cr^3+^, and Cu^2+^. Lee et al. [33] evaluated the adsorption efficiency of a modified carbon foam, reporting uptake values of 6.7, 3.8, and 6.4 mg·g^−1^ for Cr, Cu, and Ni, respectively. Li et al. [34] synthesized nitrogen-doped carbon foam loaded with nanoscale zero-valent iron in a core-shell configuration, which exhibited adsorption capacities of 1.97 mg·g^−1^ and 13.9 mg·g^−1^ for Cd^2+^ in soil and water, respectively. Despite the exceptional adsorption performance of modified carbon foams, their widespread application is hindered by high production costs and complex fabrication processes. Therefore, substituting phenol with agricultural and forestry waste (including bamboo- and shell-based waste) in the synthesis of modified carbon foam presents a critical and pragmatic solution to address these limitations.

This study aims to evaluate the removal performance of modified carbon foam, prepared by partially substituting phenol with bamboo powder waste, for Pb^2+^ and Cd^2+^ in aqueous solutions. To date, there have been no reports on the use of modified carbon foam synthesized by partially replacing phenol with whole-component bamboo powder waste liquefaction products for heavy metal adsorption. To further investigate the impact of the silane coupling agent on the properties of the carbon foam, N_2_ adsorption isotherms, SEM, FTIR, and SEM-EDS were employed to analyze the pore structure, microstructure, functional group composition, and surface elemental composition of the carbon foam before and after modification. Additionally, the influences of contact time, solution pH, initial metal concentration, and temperature on Pb^2+^ and Cd^2+^ removal were systematically examined. Finally, XPS analyses of M-CF before and after adsorption were conducted to clarify the underlying adsorption mechanisms.

## 2. Results and Discussion

### 2.1. Characterization of Two Carbon Foams

#### 2.1.1. SEM Images of U-CF and M-CF

**Figure 1:**
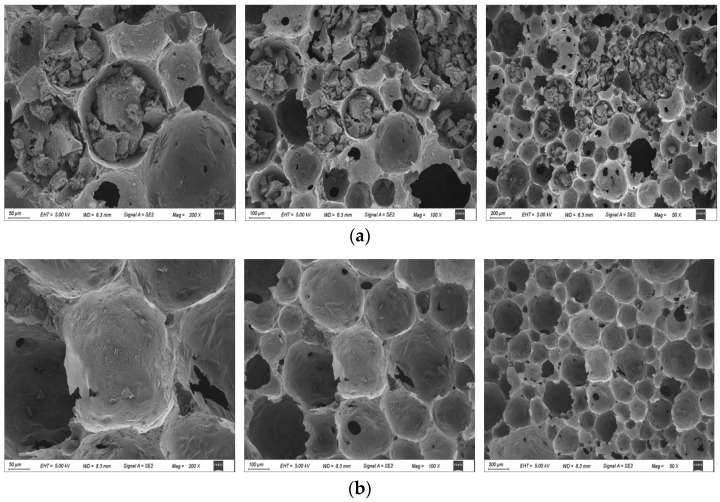
Comparative SEM images of two types of carbon foam at different magnifications: (**a**) U-CF and (**b**) M-CF.

The structural morphology of the unmodified carbon foam (U-CF) and M-CF was analyzed using SEM (see Figure 1). As shown in Figure 1a,b, both U-CF and M-CF exhibit a uniform, well-developed porous structure dominated by spherical pores. This architecture facilitates the infiltration of contaminants containing solutions onto the surface of materials, thereby increasing the contact area and enhancing adsorption capacity [35]. Compared with the intact pores in Figure 1b, the U-CF shows a small number of collapsed pores and partially damaged pore walls. In contrast, the spherical pores of the M-CF remain largely intact, indicating that the incorporation of a silane coupling agent improves resin cross-linking and reduces the brittleness of the carbon-foam pore walls. Furthermore, the optimization of the pore structure of carbon foam by KH560 may indicate that M-CF possesses superior adsorption capacity—a finding corroborated by its higher removal efficiencies for Pb^2+^ and Cd^2+^. In Table S1, M-CF achieved a Pb^2+^ removal efficiency of 90.8%, which was 25.59% higher than that of U-CF, and 33.6% higher for Cd^2+^. Mechanically, the presence of spherical pores contributes to improved mechanical performance and a higher specific surface area. Notably, both types of carbon foam exhibit relatively thin pore walls, which reduce the diffusion distance for adsorbates into the pores, potentially accelerating both adsorption and desorption rates.

#### 2.1.2. Porosity Structure Analysis of U-CF and M-CF

**Figure 2:**
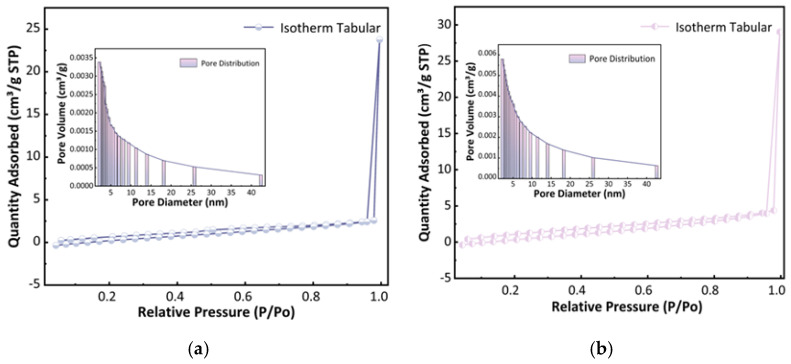
Pore size distribution profiles of the two types of carbon foam: (**a**) U-CF and (**b**) M-CF.

The pore size of carbon foam has a significant impact on its performance. As shown in Figure 2, both types of carbon foam exhibit Type IV adsorption isotherms, and Table 1 indicates that their average pore sizes range from 5.04 nm to 3.25 nm, suggesting the presence of mesoporous structures in both materials. Furthermore, Figure 1 reveals that both types of carbon foam possess uniform, spherical, thin-walled pores with diameters of approximately 25 µm, indicating the presence of macropores in U-CF and M-CF. The presence of these macropores and the mesoporous structure collectively indicate that both U-CF and M-CF possess a multilevel pore structure. Furthermore, the sharp increase in adsorption at relative pressures of 0.9–1.0 in Figure 2 further confirms the existence of macroporous structures [36]. These macropores primarily originate from the foaming process of phenolic resin and typically endow carbon foam with faster adsorption rates and better regeneration performance. However, larger pore sizes and thinner pore walls impede the formation of micropores. The t-Plot external surface area is very close to the BET test results (Table 1), indicating that the micropore content in both carbon foams is extremely low, further confirming that larger pore sizes are unfavorable for micropore formation. After modification, the total pore volume of the carbon foam increased slightly from 0.022 cm^3^/g to 0.032 cm^3^/g, while the specific surface area increased by 27.46%. These changes demonstrate that the addition of the modifier effectively regulated the pore structure development of the carbon foam. Upon incorporation of KH560, its methoxy groups (–OCH_3_) undergo hydrolysis under acidic conditions, generating reactive silanol groups (–Si–OH) and releasing methanol as a byproduct. These silanol groups subsequently engage in condensation reactions with hydroxyl groups (–OH) on the bamboo powder surface, forming stable Si–O–C covalent bonds and achieving chemical anchoring of KH560 [37]. Additionally, the γ-glycidyloxypropyl group (–CH_2_–CH(O)–CH_2_–) in KH560 exhibits electrophilic properties. Under alkaline conditions with NaOH and heat, the epoxy ring undergoes nucleophilic addition with phenolic hydroxyl groups in the phenolic resin, leading to ring-opening and covalent bond formation with the resin. Consequently, KH560 acts as a molecular bridge between bamboo powder and the resin, enhancing interfacial bonding performance.

#### 2.1.3. Infrared Spectral Analysis of Carbon Foam

**Figure 3:**
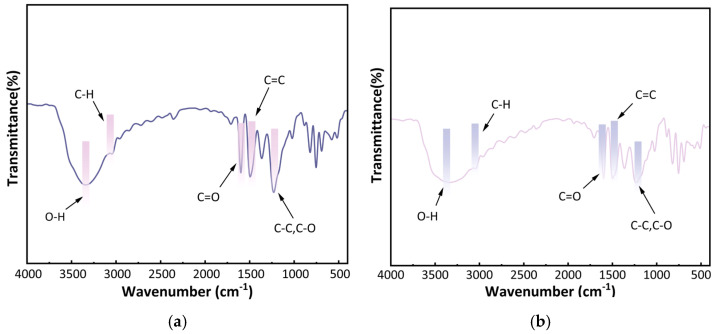
Infrared spectra of the two carbon foam samples: (**a**) U-CF and (**b**) M-CF.

Figure 3 presents the infrared spectral curves (FTIR) of the carbon foam before and after modification. By comparing the spectra, it is evident that the spectral compositions of U-CF and M-CF are largely similar, indicating that the functional group structure of the carbon foam remains essentially unchanged following modification. Further examination of Figure 3 reveals that both U-CF and M-CF exhibit a prominent vibrational peak near 3357 cm^−1^, which is primarily attributed to the O–H stretching vibrations of alcoholic and phenolic hydroxyl groups [38,39]. The peak at 2943 cm^−1^ corresponds to the asymmetric stretching vibrations of methylene C-H bonds. The peak near 1613 cm^−1^ is associated with the stretching vibrations of the C=O bond [40], while the peak at 1473 cm^−1^ is attributed to the stretching vibrations of the aromatic ring skeleton C=C bond. The peak appearing around 1217 cm^−1^ results from the stretching vibrations of C-C and C-O bonds [41,42]. Compared to U-CF, M-CF shows a slight change in the peak near 3357 cm^−1^. This change is due to the condensation reaction between the hydrolysis products of the KH560 and the hydroxyl groups, resulting in the formation of Si-O-C bonds [43]. This reaction consumes some of the hydroxyl groups. Additionally, the symmetric vibration peak of the Si-O bond at 817 cm^−1^ verifies the successful silanization of the resin, indicating that silicon has been effectively incorporated into the modified resin structure.

#### 2.1.4. SEM-EDS Analysis

**Figure 4:**
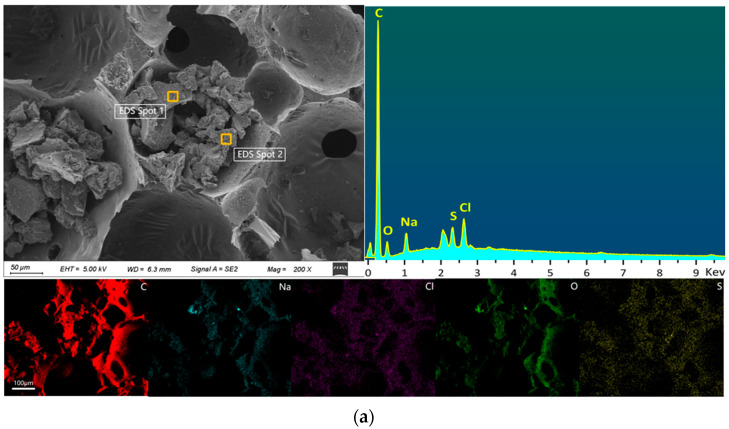

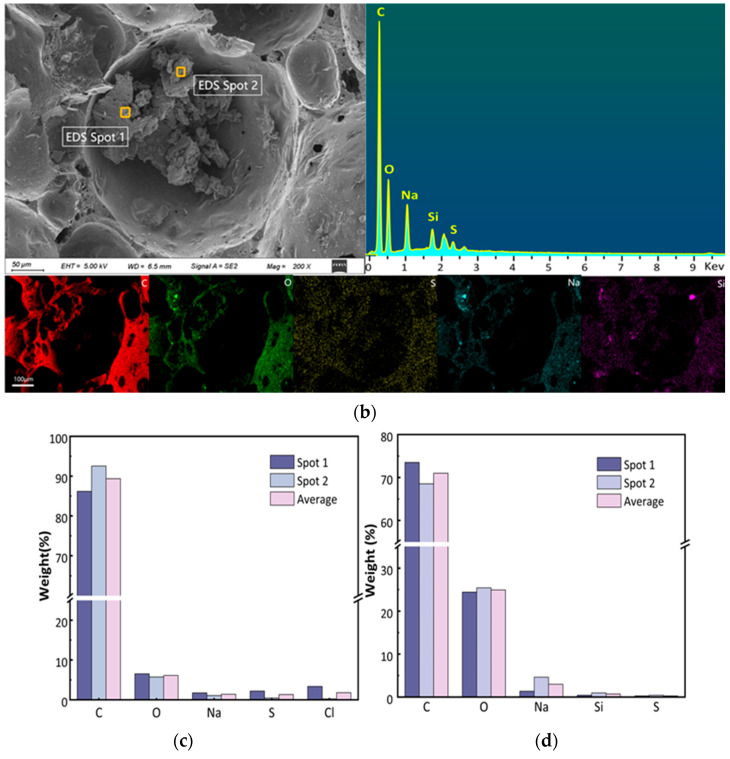
SEM-EDS images of the two phenolic resin-based carbon foams (**a**) U-CF and (**b**) M-CF, and (**c**) major elemental composition of U-CF and (**d**) major elemental composition of M-CF. Note: EDS Spot 1 denotes SEM-EDS scanning point 1, representing the elemental analysis performed by the EDS at the first selected location on the sample surface, while EDS Spot 2 indicates SEM-EDS scanning point 2.

Scanning electron microscopy coupled with energy-dispersive X-ray spectroscopy (SEM-EDS) is a widely used technique for characterizing surface morphology and elemental distribution of materials. SEM acquires microstructural and morphological information by scanning the sample surface with an electron beam, while EDS enables qualitative and semi-quantitative elemental analysis through detection of characteristic X-rays. The SEM-EDS results in Figure 4 indicate that, compared to U-CF, M-CF exhibits significant alterations in surface elemental composition and content. The C content decreases from 89.36% in U-CF to 71.01% in M-CF, likely due to the detachment of carbon atoms bonded to carboxyl groups during the modification process, where active functional groups react with hydroxyl groups on the carbon foam surface [44]. Additionally, the introduction of KH560 may have diluted the carbon phase by forming Si–O–C bonds. The primary sources of carbon are the carbon foam matrix and carbonate ions (CO_3_^2−^) present in the particles. Conversely, the O content increases markedly from 6.11% in U-CF to 24.93% in M-CF, indicating that oxygen participates in chemical reactions during modification. Additionally, trace amounts of Na are detected in both U-CF and M-CF, possibly stemming from the NaOH used in the foaming process. Notably, Si is uniquely present in M-CF, attributable to the incorporation of a silane coupling agent. The Cl content varies significantly between two selected points within the same region, with values of 3.37% and 0.23%, respectively; this indicates a non-uniform distribution of elements on the carbon foam surface.

### 2.2. Effect of pH

**Figure 5:**
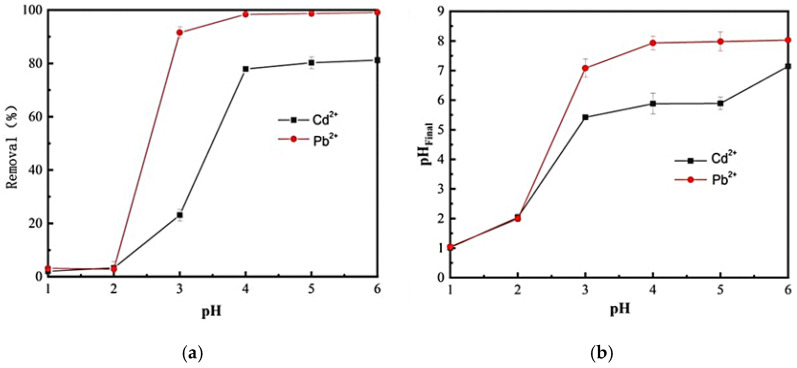
Effect of solution pH on the removal of Pb^2+^ and Cd^2+^ by M-CF. (**a**) Removal efficiency of Pb^2+^ and Cd^2+^; by M-CF under different pH conditions; (**b**) Variation trend of final solution pH after adsorption of Pb^2+^ and Cd^2+^ by M-CF at different initial pH values. Initial concentrations of Pb^2+^ and Cd^2+^ were both 150 mg·L^−1^, with adsorption time: 24 h, adsorption temperature: 25 °C, V(Pb^2+^)/m(M-CF) = 500, and V(Cd^2+^)/m(M-CF) = 100.

The pH of a solution is a critical parameter in adsorption processes, as it significantly influences the solubility and speciation of heavy metal ions [45]. In this study, Pb^2+^ and Cd^2+^ begin to precipitate at pH values of 6.2 and 8.8, respectively, with further increases in pH leading to hydroxide formation and subsequent precipitation [46]. To avoid pre-adsorption precipitation of Pb^2+^ and Cd^2+^, this work analyzed the removal efficiency of carbon foam for these ions within a pH range of 1–6. The final pH of the solution after adsorption was also measured, with results presented in Figure 5.

As illustrated in Figure 5a, the removal rates of Pb^2+^ and Cd^2+^ by M-CF do not significantly change when the solution pH is below 2. However, as the pH increases further, there is a marked increase in the removal rates of both Pb^2+^ and Cd^2+^. At a pH of 4, the uptake rates for Pb^2+^ and Cd^2+^ are 98.2% and 79.8%, respectively. Analysis of the post-adsorption solution pH_Final_ in Figure 5b reveals that the addition of M-CF does not significantly alter the solution pH under low initial pH conditions. However, when the initial pH exceeds 3, the pH_Final_ of the solution containing M-CF rapidly increases; at pH 4, the final pH of Pb^2+^ and Cd^2+^ solutions approaches 8 and 6, respectively. This pH elevation reduced H^+^ concentration, weakening competitive adsorption between protons and Cd^2+^/Pb^2+^, while promoting the transformation of Pb^2+^ and Cd^2+^ into hydrolyzed species such as hydroxoplumbyl ion [Pb(OH)]^+^ [47], hydroxocadmylyl ion [Cd(OH)]^+^, Pb (OH)_2_, and Cd (OH)_2_, thereby enhancing removal efficiency. Based on Figure 5, an optimal pH range of 4–6 is recommended for effective adsorption.

### 2.3. Kinetic Analysis

**Figure 6:**
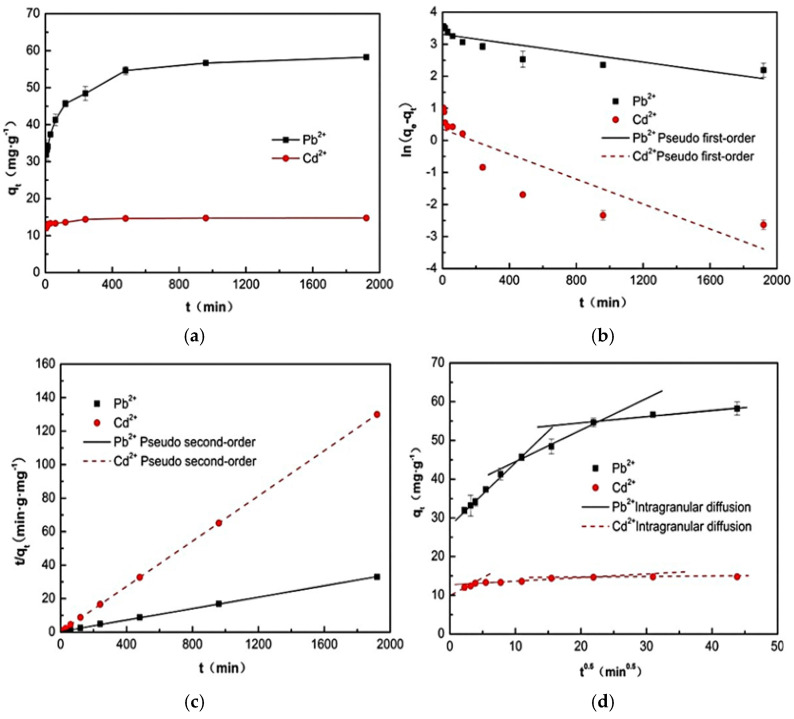
(**a**) Effect of adsorption time on the removal rate, (**b**) pseudo-first-order kinetics, (**c**) pseudo-second-order kinetics, and (**d**) intra-particle diffusion model. Adsorption conditions: initial concentration of Pb^2+^ and Cd^2+^: 150 mg·L^−1^, pH 4, V(Pb^2+^)/m(M-CF) = 500, and V(Cd^2+^)/m(M-CF) = 100.

As evidenced by the comparative curves in Figure 6a, the adsorption of Pb^2+^ and Cd^2+^ by M-CF proceeds through three distinct phases: a rapid initial phase (Pb^2+^: 0–120 min and Cd^2+^, 0–60 min), a slower intermediate phase (Pb^2+^: 120–960 min and Cd^2+^: 60–480 min), and equilibrium attainment at 960 min and 480 min. Analysis of Figure 6b,c, along with the data in Table 2, reveals that the pseudo-second-order kinetic model provides a superior fit to the experimental data, with correlation coefficients (R^2^) of 0.9994 and 0.9990 for Pb^2+^ and Cd^2+^, respectively. These values significantly exceed those obtained using the pseudo-first-order model. Furthermore, the theoretical equilibrium adsorption capacities derived from the pseudo-second-order model (58.56 mg·g^−1^ for Pb^2+^ and 14.79 mg·g^−1^ for Cd^2+^) closely align with the experimental values (58.24 mg·g^−1^ for Pb^2+^ and 14.75 mg·g^−1^ for Cd^2+^), confirming that the adsorption process adheres to pseudo-second-order kinetics. This suggests that the adsorption mechanism is predominantly governed by the physicochemical properties of the carbon foam [48].

The intra-particle diffusion model, as presented in Figure 6d, reveals three distinct stages for both Pb and Cd adsorption. The initial stage corresponds to surface adsorption onto M-CF. The second stage involves gradual intra-particle or intra-pore diffusion [49], which constitutes the rate-limiting step due to its prolonged duration. The final stage represents equilibrium adsorption. Notably, the W-M intra-particle diffusion model does not yield a linear plot passing through the origin, indicating that intra-particle or intra-pore diffusion is not the sole rate-controlling mechanism [50]. Instead, film diffusion also plays a significant role in regulating the adsorption kinetics.

### 2.4. Adsorption Isotherm Analysis

**Figure 7:**
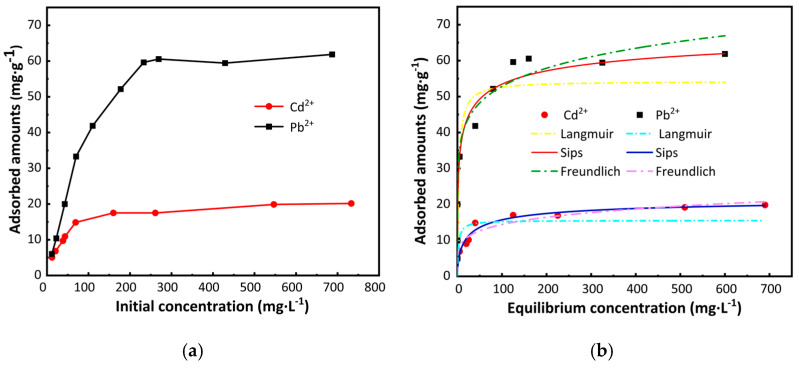
(**a**) Effect of initial Pb^2+^ and Cd^2+^ concentrations on removal rates; (**b**) Langmuir, Freundlich, and Sips isotherm Models.

The concentration of heavy metal ions significantly influences the adsorption capacity of carbon foam, with the effect generally varying with concentration changes. As shown in Figure 7a, the adsorption capacities of M-CF for Pb^2+^ and Cd^2+^ in water initially increased rapidly with rising concentrations of both ions, followed by a gradual deceleration. Upon reaching initial concentrations of 230 mg·L^−1^ for Pb^2+^ and 160 mg·L^−1^ for Cd^2+^, further increases in ion concentration resulted in the adsorption capacities for both Pb^2+^ and Cd^2+^ approaching equilibrium. To better illustrate the relationship between the equilibrium adsorption capacities of the carbon foam for Pb^2+^ and Cd^2+^ and their respective equilibrium concentrations, the experimental data were fitted to the Langmuir, Freundlich, and Sips isotherms. The fitting results and corresponding parameters are presented in Figure 7b and Table 3.

Generally, the Langmuir model assumes a homogeneous adsorbent surface with uniform energy at active adsorption sites, resulting in monolayer adsorption [51]. In contrast, the Freundlich model describes adsorption on energetically heterogeneous surfaces, while the Sips model represents a hybrid of the two aforementioned frameworks [52]. In Table 3, the Sips isotherm model yielded determination coefficients of 0.9609 and 0.9597 for Pb^2+^ and Cd^2+^, respectively. These values are higher than those obtained from the Langmuir and Freundlich isotherm models, indicating that the Sips isotherm model more accurately describes the relationship between the equilibrium concentrations of Pb^2+^ and Cd^2+^ and their corresponding adsorption capacities. This suggests that the adsorption of Pb^2+^ and Cd^2+^ ions by the carbon foam continues beyond the first layer [53]. As shown in Table 3, the maximum adsorption capacities provided by the Sips isotherm model are 61.59 mg·g^−1^ for Pb^2+^ and 22.15 mg·g^−1^ for Cd^2+^, which are close to the experimentally obtained values (62.84 mg·g^−1^ and 24.30 mg·g^−1^, respectively). This indicates that the Sips model can accurately predict the actual adsorption capacities of M-CF for these two heavy metals. At low concentrations of Pb^2+^ and Cd^2+^, multilayer adsorption occurs, while at higher concentrations of Pb^2+^ and Cd^2+^, monolayer adsorption takes place [54].

### 2.5. Adsorption Mechanism Analysis

Investigating the mechanism of the adsorption process is crucial for further modulating the adsorption performance of M-CF. Research by Lee et al. [31] on the adsorption of heavy metal ions using phenolic resin-based carbon foam revealed that the adsorption of Pb^2+^ and Cu^2+^ from water primarily involves the formation of sulfide precipitates through a chemical adsorption process. Kinetic and isotherm studies indicated that the adsorption of Pb^2+^ and Cd^2+^ was a combined effect of both physisorption and chemisorption. While chemical adsorption may involve precipitation, it could also involve complexation between the surface functional groups of carbon foam and Pb^2+^/Cd^2+^, which requires further analysis for confirmation. Currently, FTIR technology is primarily used to observe significant changes or shifts in the relevant functional groups before and after adsorption to determine the presence of complexation. However, due to the extremely low concentration of heavy metal ions in the adsorption process, obtaining ideal results with FTIR is challenging [55].

**Figure 8:**
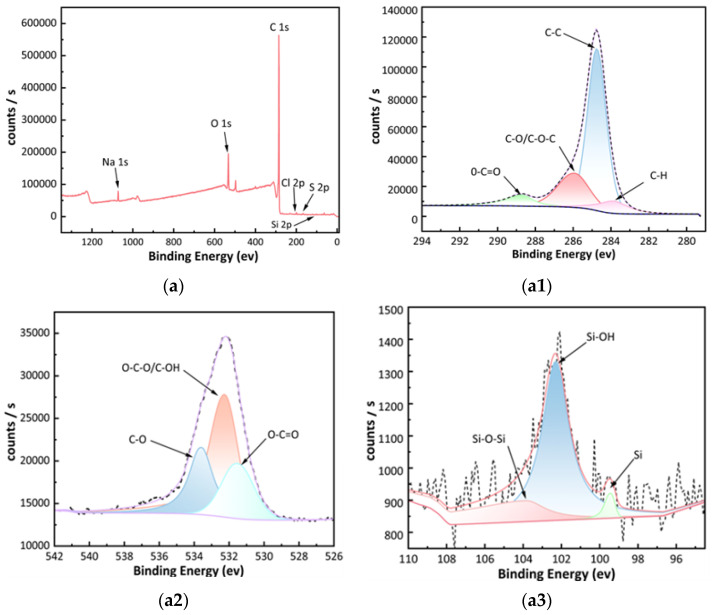

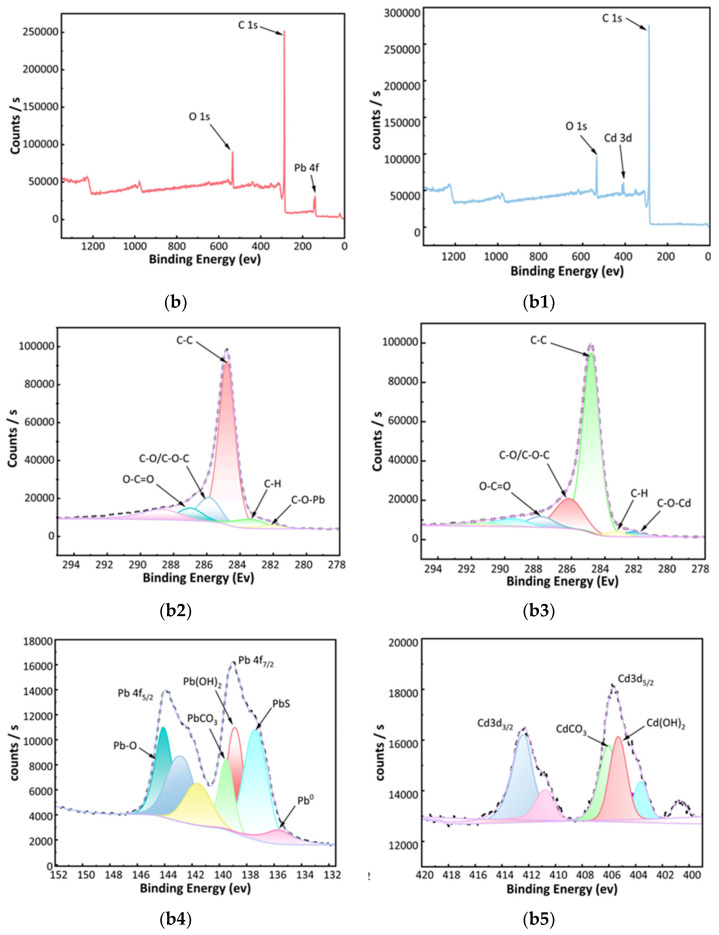
XPS characterization of M-CF before and after heavy metal adsorption. (**a**) Full spectrum of M-CF before adsorption. (**a1**,**a2**,**a3**) show high-resolution spectra of C1s, O1s, and Si2p from carbon foam, respectively. (**b**,**b1**) show the full spectra of Pb4f and Cd3d for M-CF after heavy metal ion adsorption, respectively; (**b2**,**b3**,**b4**,**b5**) present the spectra of C1s-Pb, C1s-Cd, Pb4f, and Cd3d after adsorption, respectively.

In light of the foregoing considerations, to further elucidate the adsorption mechanism of M-CF for Pb and Cd, XPS was employed to analyze the carbon foam before and after adsorption (Figure 8). Figure 8(a1,a2,a3) present the C1s, O1s, and Si2p spectra of M-CF, respectively. Prior to adsorption, the C1s spectrum of M-CF exhibited a peak at 283.39 eV corresponding to C-H bonds, and a peak at 284.75 eV indicative of C-C bonds. Further peaks were observed at 286.0 eV and 288.93 eV, attributed to C-O/C-O-C and O-C=O functional groups, respectively [56,57,58]. Analysis of the four peaks in (a1) revealed that C-C bonds comprised the highest relative content (66.71%), followed by C-O/C-O-C (19.17%), while C–H bonds accounted for the lowest proportion at merely 3.68%. The O1s spectrum in (a2) primarily centered around 532 eV [59], with constituent peaks at 531.50 eV (O-C=O), 532.26 eV (O-C-O/C-OH), and 533.58 eV (C-O). Furthermore, the presence of three Si peaks, characteristic of the modification process, was confirmed near 102 eV [60], specifically at 102.3 eV (Si-OH) and 103.87 eV (Si-O-Si). The identification of these novel functional groups confirms successful incorporation of the modifying agent into the resin, consistent with the FTIR analysis presented in Figure 3.

A comparison of Figure 8(a1) with Figure 8(b2,b3) reveals that the C-C bond content of Pb-C1s and Cd-C1s decreased from 66.71% to 63.66% and 61.75%, respectively, after the adsorption of heavy metals. This indicates that C-C bonds were partially consumed during the adsorption of heavy metals by M-CF, likely due to the chelation reaction between Pb^2+^ and Cd^2+^ with hydroxyl and carboxyl groups on the M-CF surface, which indirectly weakened the C-C bonds. Alternatively, the deposition of Pb and Cd on the carbon surface may have covered some of the carbon, leading to a decrease in the C-C bond signal. Additionally, the content of C-O/C-O-C bonds decreased from 19.17% to 16.52% and 12.22%, respectively, which may be associated with the formation of Pb-O chelates. During this process, electrons can transfer from the oxygen atom to the lead ion surface [61]. Furthermore, the O-C=O content of Pb-C1s decreased by 3.98% compared to before adsorption, which is higher than the 0.18% decrease observed for Cd-C1s. This difference may be attributed to the larger radius of Pb^2+^, which results in stronger chelation with O-C=O. New peaks appeared at 282.33 eV and 282.16 eV for Pb-C1s and Cd-C1s, respectively, corresponding to C-O-Pb [62] and C-O-Cd. This further indicates that the chelation action of oxygen-containing functional groups played a role in the adsorption process, consistent with the findings of Lee et al. [31].

Analysis of Figure 8(b4) reveals that the Pb species are characterized by two distinctive peaks: Pb4f7/2 at 139.08 eV and Pb4f5/2 at 143.98 eV [63]. The Pb4f7/2 peak can be deconvoluted into four component peaks: Pb^0^ at 135.70 eV, PbS at 137.35 eV, Pb (OH)_2_ at 138.83 eV, and PbCO_3_ at 139.50 eV. Among these species, PbS, Pb (OH)_2_, and PbCO_3_ account for 47.41%, 33.10%, and 19.49% of the total Pb content, respectively, indicating that Pb is primarily removed by the carbon foam in these three chemical forms. Similarly, the Cd species also exhibit two main peaks. As shown in Figure 8(b5), the Cd3d5/2 peak can be deconvoluted into two component peaks at 405.30 eV and 406.03 eV, corresponding to Cd (OH)_2_ (49.66%) and CdCO_3_ (50.34%), respectively [64,65]. These results demonstrate that the Pb^2+^ and Cd^2+^ ions removed by M-CF primarily exist as hydroxide and carbonate precipitates. Unlike Cd, the removal of Pb also involves the formation of sulfide precipitation. The formation of hydroxides is likely attributed to the increase in pH_Final_ following the addition of carbon foam, while the generation of carbonates may result from either direct reactions between the metal ions and CO_3_^2−^ or the transformation of hydroxides to carbonates due to the lower solubility of metal carbonates compared to their hydroxide counterparts under dissolution-precipitation equilibrium conditions. In conclusion, the removal of Cd^2+^Pb^2+^ and Pb^2+^ from aqueous solutions by M-CF involves both physical adsorption facilitated by its unique multi-porous structure and chemical precipitation through the formation of hydroxides, sulfides, and carbonates. The corresponding reaction equations are presented in Equations (1)–(3).

(1) $$M^{2+}+{2OH}^{-}\to {M\left(OH\right)}_{2}$$

(2) $$M^{2+}+{CO}_{3}^{2-}\to {MCO}_{3}$$

(3) $${M\left(OH\right)}_{2}+{CO}_{3}^{2-}\to M{CO}_{3}^{2-}+{OH}^{-}$$

Note: In Equations (1)–(3), M represents Pb^2+^ and Cd^2+^.

## 3. Materials and Methods

### 3.1. Materials

Uncrushed giant dragon bamboo (*Dendrocalamus sinicus*) was purchased from the Southwest Wood Processing Factory (Kunming, China). Phenol, formaldehyde, NaOH, sulfuric acid, activated carbon, n-hexane, and the silane coupling agent were all analytical-grade reagents supplied by the National Pharmaceutical Group (Beijing, China). Analytical-grade reagents Cd(NO_3_)_2_·4H_2_O and Pb(NO_3_)_2_ were purchased from Tianjin Chemical Reagent Co., Ltd, (Tianjin, China) and Beijing Chemical Factory (Beijing, China), respectively. The 1000 mg·L^−1^ Pb^2+^ and Cd^2+^ standard solutions were obtained from the National Steel Research Institute.

### 3.2. Preparation of U-CF and M-CF

#### 3.2.1. Preparation of Unmodified Phenolic Resin Foam

Giant dragon bamboo waste, after the removal of large particulate impurities, was dried in an oven at 102 °C. The dried bamboo powder was then finely ground and sieved through an 80-mesh screen. Subsequently, 30 g of the resulting bamboo powder was accurately weighed and mixed with phenol at a mass ratio of 3:7. The mixture was transferred into a reaction vessel and heated to 140 °C, followed by the dropwise addition of 5% H_2_SO_4_ (by weight of phenol) as a catalyst. After complete addition, the reaction was maintained for 2 h under stirring. The resulting product was then cooled and stored appropriately. For further utilization, the cooled product in a three-necked flask was placed in a water bath and heated to 60 °C. Sodium hydroxide (NaOH), equivalent to 7% of the mass of phenol, was added, followed by the addition of a 37% formaldehyde solution (with a molar ratio of phenol to formaldehyde of 1:1.8). After reacting for 0.5 h, the temperature was raised to 80 °C and maintained for 2 h. Upon completion of the reaction, the three-neck flask was removed and cooled using distilled water. The pH was adjusted to 7–8 with dilute hydrochloric acid. Finally, the reaction mixture was subjected to rotary evaporation (RE-2000, Shanghai Yalong Biochemical Instrument Factory, Shanghai, China) to achieve a resin viscosity of 500–600 mPa·s.

For unmodified foam preparation, 30 g of the concentrated resin was sequentially mixed with 4% Tween-80, 5% curing agent (50% sulfuric acid solution, 1:1 by volume), and 6% foaming agent. The resin mixture containing the curing agent, foaming agent, and surfactant was homogenized using a high-speed mixer at 1000 rpm until a silvery-gray appearance was obtained. The homogeneous mixture was then transferred into a foaming mold and cured in an 80 °C oven for 0.5 h. The unmodified foam was obtained after cooling to room temperature.

#### 3.2.2. Preparation of Modified Phenolic Resin Foam

The liquefaction process for the modified phenolic foam was identical to that of the unmodified foam. The resinification stage, however, was performed differently. The synthesis was conducted in a three-neck flask equipped with a mechanical stirrer and a reflux condenser. Initially, phenol and formaldehyde were added at room temperature at a molar ratio of 1:1.8. The system was then heated to 40 °C, at which point NaOH, equivalent to 7% of the phenol mass, was introduced as a catalyst. Subsequently, the temperature was raised to 60 °C at a controlled rate of 2 °C/min and maintained for 0.5 h to complete the reaction. Subsequently, 4.1 wt% silane coupling agent was added to the reaction system, followed by heating to 95 °C at 3 °C/min and maintaining this temperature for 50 min. The reaction mixture was then cooled using chilled distilled water and neutralized with 6 mol·L^−1^ hydrochloric acid to achieve a pH between 6 and 7. The modified resin was concentrated by rotary evaporation to attain a target viscosity range of 500–700 mPa·s. For modified foam preparation, 30 g of the modified resin was sequentially mixed with 4 wt% Tween-80, 5 wt% curing agent, and 6 wt% foaming agent. The mixture was homogenized using a high-speed mixer at 1000 rpm until complete uniformity was achieved, then transferred to a foaming mold and cured in an 80 °C oven for 0.5 h. The final modified phenolic foam was obtained after cooling to ambient temperature.

#### 3.2.3. Preparation of Foaming Agent and Carbonization of Resin Foam

To prepare the foaming core, 3 g of wood-based activated carbon with a particle size range of 80–150 μm was immersed in an excess of n-hexane for 20 min. Following vacuum filtration, the activated carbon was sealed in a high-pressure autoclave and heated in an oven at 140 °C for 2 h. After naturally cooling, the resulting transplanted core foaming agent exhibited an n-hexane loading of 27.1 wt%.

**Figure 9:**
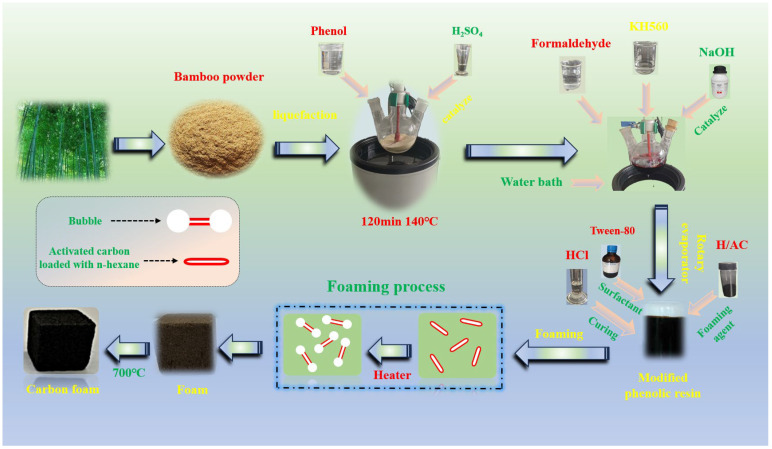
Fundamental preparation process of phenolic-based porous resin carbon foam.

Both the modified and unmodified phenolic foams were placed in a vacuum tube furnace for carbonization. The temperature was first increased to 500 °C at a heating rate of 5 °C/min, followed by further heating to 700 °C at a reduced rate of 2 °C/min. The samples were maintained at 700 °C for 2 h to produce the carbonized foams, designated as U-CF and M-CF, respectively. The preparation process for the modified carbon foam is illustrated in Figure 9. In Figure 9, n-hexane-loaded activated carbon particles are observed as slender, tube-like structures (labeled in the figure as a red elongated ellipse enclosing a white region). As n-hexane vaporizes on the activated carbon, the resulting gas accumulates along the cracks and converges towards the particle ends. At these ends, a lower free energy barrier facilitates the preferential formation of gas bubbles, which are depicted as spherical bubbles attached to both ends of the elongated elliptical particle. With increased n-hexane evaporation, the pressure generated by the expanding gas overcomes the free energy barrier of the surrounding resin, displacing it and forming cavities. Due to the surface tension of the surrounding resin, these cavities exist within the resin as spherical bubbles, which is the morphology that minimizes their surface energy.

### 3.3. Adsorption Experiments and Analytical Methods

Stock solutions (1000 mg·L^−1^) of Pb^2+^ and Cd^2+^ were prepared by dissolving Pb(NO_3_)_2_ and Cd(NO_3_)_2_·4H_2_O in deionized water, respectively, followed by stepwise dilution. The concentrations of both Pb^2+^ and Cd^2+^ ions were set at 150 ppm. During this process, the pH of the solutions was adjusted using 0.1 mol·L^−1^ NaOH.

Precisely 0.1 g of carbon foam was weighed and added to a polyethylene centrifuge tube. Pb^2+^ and Cd^2+^ solutions were then added, maintaining a defined liquid-to-solid ratio (a solution volume to carbon foam mass of 100:1). The resulting mixture was shaken at 120 rpm in a horizontal, temperature-controlled water bath shaker. Subsequently, the mixture was filtered through a 0.22 μm syringe filter, and the filtrate and residue were collected separately. The concentrations of residual Pb^2+^ and Cd^2+^ in the filtrate were determined using an Inductively Coupled Plasma Optical Emission Spectrometer (VISTA-MPX, Varian, CA, USA). Removal efficiency (R) and adsorption amount at adsorption equilibrium (q_e_) were calculated using Equations (4) and (5), respectively. XPS analysis was performed on the M-CF residue to investigate the removal mechanism. To minimize experimental error, each batch of experiments in this study was repeated 3 times, and the results are reported as mean values.

(4) $$R=\frac{C_{0}-C_{e}}{C_{0}}\times 100\%$$

(5) $$q_{e}=\frac{\left(C_{0}-C_{e}\right)V}{m}$$

In the given equation, C_0_ and C_e_ are the initial solution concentration and the solution concentration at adsorption equilibrium, respectively (mg·L^−1^); q_e_ is the adsorption amount at adsorption equilibrium (mg·g^−1^); (V/m) is the liquid-to-solid ratio, expressed in mL·g^−1^; and R denotes the removal rate of heavy metal ions (%).

The experimental data were fitted using Langmuir, Freundlich, and Sips isotherm models.

(6) $$q_{e}=\frac{q_{m}K_{L}c_{e}}{1+K_{L}c_{e}}$$

(7) qe=KFce1nF

(8) $$q_{e}=\frac{q_{m}K_{S}c_{e}^{1/n_{s}}}{1+K_{S}c_{e}^{1/n_{s}}}$$

where q_m_ is the monolayer saturation capacity (mg·g^−1^); q_e_ is the adsorption amount at adsorption equilibrium (mg·g^−1^); K_L_ is the Langmuir equilibrium constant; and K_F_ and K_S_ are empirical parameters, respectively. The equilibrium concentration was represented by c_e_ (mg·L^−1^); n_S_ and n_F_ are constants associated with the Sips and Freundlich models, respectively.

Kinetic fitting was performed using both kinetic models and the Weber–Morris (W-M) intra-particle diffusion model. The corresponding fitting equations are shown as Equations (9)–(11), respectively.

(9) $$\ln\left(q_{e}-q_{t}\right)=\ln q_{e}-k_{1}t$$

(10) $$\frac{t}{q_{t}}=\frac{1}{k_{2}q_{e}^{2}}+\frac{t}{q_{e}}$$

(11) $$q_{t}=k_{p}t^{\frac{1}{2}}+I$$

Equation (9) corresponds to the pseudo-first-order kinetics. Equation (10) corresponds to the pseudo-second-order kinetics. Equation (11) corresponds to the W-M intra-particle diffusion model. q_e_ is the adsorption amount at adsorption equilibrium (mg·g^−1^); k_1_ is the pseudo-first-order adsorption rate constant (min^−1^); k_2_ is the pseudo-second-order adsorption rate constant (g·mg^−1^·min^−1^); q_t_ indicates the amount of adsorption (mass of solute adsorbed per unit of adsorbent at time t) in mg·g^−1^; k_p_ is the intra-particle diffusion rate constant (mg·g^−1^·min^0.5^); and I is a constant related to the boundary layer thickness (mg·g^−1^).

Figure 9 was created using Microsoft PowerPoint (2021 version), while all other images were generated using Origin 2024 software.

### 3.4. Characterization Test

The specific surface area and pore size distribution of the carbon foam samples were analyzed using a fully automated surface area and porosity analyzer (Micromeritics, GA, USA). The samples were pretreated under vacuum at −195.850 °C for 12 h, followed by nitrogen adsorption–desorption measurements conducted at 77 K using liquid nitrogen. Based on the obtained adsorption–desorption isotherms, the total specific surface area of the material was calculated using the BET method. X-ray photoelectron spectroscopy (K-Alpha+, Thermo Fisher Scientific (Waltham, MA, USA)) was employed to analyze the elemental composition and chemical states of the carbon foam after heavy metal adsorption. A monochromatic Al Kα X-ray source was utilized, and survey scans were conducted with a pass energy of 100 eV and a step size of 1 eV. The spectra were calibrated by referencing the C1s peak of adventitious carbon at 284.8 eV. Scanning electron microscopy with energy-dispersive X-ray spectroscopy (Sigma300, ZEISS, Jena, Germany) was used to acquire information on the sample’s cross-sectional morphology, particle size, and other microstructural features. An accelerating voltage of 10 kV was employed, and samples were sputter-coated with gold for 45 s prior to analysis. Fourier transform infrared spectroscopy (TENSOR27, BRUKER, Ettlingen, Germany) was employed to identify the vibrational absorption bands of functional groups present in the carbon foam samples. The experimental parameters were as follows: samples were prepared as KBr pellets, spectra were recorded at a resolution of 4 cm^−1^ over a spectral range of 4000–400 cm^−1^, and 32 scans were accumulated.

## 4. Conclusions

In this study, a novel modified carbon foam was successfully synthesized utilizing bamboo powder waste as a partial phenol substitute, followed by modification with a silane coupling agent and foaming using H/AC. The carbon foam was characterized, and its adsorption performance for aqueous Pb^2+^ and Cd^2+^ was analyzed along with investigation of the removal mechanisms. Results demonstrated that M-CF exhibited a porous structure with spherical cavities and effective adsorption capacity for Pb^2+^ and Cd^2+^ in aqueous solutions. The adsorption process followed the Sips isotherm model and pseudo-second-order kinetics, with maximum adsorption capacities of 22.15 mg·g^−1^ for Cd^2+^ and 61.59 mg·g^−1^ for Pb^2+^. The removal mechanisms of aqueous Pb^2+^ and Cd^2+^ by M-CF primarily involved three aspects: complexation, physical adsorption, and chemical precipitation, with the precipitates predominantly consisting of hydroxides, carbonates and PbS. These findings confirm the significant potential of M-CF for the removal of Pb^2+^ and Cd^2+^ from aqueous solutions, providing scientific reference for the development of advanced heavy metal adsorbent materials.

## Figures and Tables

**Table 1:** Fundamental pore structure parameters of U-CF and M-CF.

	S_BET_ (m^2^/g)	P_Total_ (cm^3^/g)	P_size_ (nm)	S_t_ (m^2^/g)
U-CF	3.35 ± 0.27	0.022 ± 0.015	5.04 ± 1.28	4.30 ± 1.48
M-CF	4.27 ± 0.11	0.032 ± 0.005	3.25 ± 0.25	5.94 ± 0.32

Note: S_BET_ denotes the BET surface area; P_Total_ represents the total pore volume; P_size_ indicates the average pore size; and S_t_ corresponds to the t-Plot external surface area.

**Table 2:** Kinetic fitting parameters.

	Pseudo-First-Order			Pseudo-Second-Order			Intra-Particle Diffusion			
	k_1_	q_e_	R^2^	k_2_	q_e_	R^2^	Phase	k_p_	I	R^2^
Pb^2+^	7.2 × 10^−4^	27.33	0.7612	7.5 × 10^−4^	58.56	0.9994	1	1.62	28.21	0.9956
							2	0.829	36.22	0.9855
							3	0.162	51.31	0.9706
Cd^2+^	2.0 × 10^−3^	1.42	0.7708	1.5 × 10^−2^	14.79	0.9990	1	0.606	10.66	0.9214
							2	0.092	12.72	0.9462
							3	0.005	14.57	0.8414

**Table 3:** Fitting parameters of the three isotherm models.

Heavy Metal Ions	Langmuir	Freundlich	Sips
Pb^2+^	q_m_ = 54.28, K_L_ = 0.31, R^2^ = 0.6405	K_F_ = 28.79, n_F_ = 7.58, R^2^ = 0.8554	q_m_ = 61.59, K_S_ = 0.60, n_s_ = 1.83, R^2^ = 0.9609
Cd^2+^	q_m_ = 19.46, K_L_ = 0.05, R^2^ = 0.8165	K_F_ = 6.05, n_F_ = 5.30, R^2^ = 0.8260	q_m_ = 22.15, K_S_ = 0.14, n_s_ = 1.62, R^2^ = 0.9597

## Data Availability

The data of this study are available from the corresponding author upon reasonable request.

## Supplementary Materials

The following supporting information can be downloaded at: https://www.mdpi.com/article/10.3390/ijms26157302/s1.
