# Supplementary material for: Adsorption of Pb2+ and Cd2+ from Aqueous Solutions by Porous Carbon Foam Derived from Biomass Phenolic Resin
Source: Int J Mol Sci. 2025 Jul 28;26(15):7302. doi: 10.3390/ijms26157302 (PMC12346760; doi:10.3390/ijms26157302)
Supplement: Supplementary file 1 [file ijms-26-07302-s001.zip › Table S1/Table S1.pdf]

Table S1 Removal rates of lead and cadmium ions by carbon foam before and after modification

	Removal rate (%)	
	Pb^{2+}	Cd^{2+}
U-CF	72.3±1.4	55.1±2.7
M-CF	90.8±2.1	73. 6±1.9

Note: concentration of Pb^{2+} and Cd^{2+} = 120 ppm, pH = 4.00, temperature = 25 °C, adsorption time = 14 h, liquid-to-solid ratio = 100:1.
